# Coenzyme Q10: A Biomarker in the Differential Diagnosis of Parkinsonian Syndromes

**DOI:** 10.3390/antiox12122104

**Published:** 2023-12-12

**Authors:** Tereza Bartošová, Jiří Klempíř, Hana Hansíková

**Affiliations:** 1 Neurology and Center of Clinical Neuroscience, First Faculty of Medicine, Charles University and General University Hospital in Prague, 121 08 Prague, Czech Republic; tereza.bartosova2@vfn.cz (T.B.); jiri.klempir@vfn.cz (J.K.); 2 Laboratory for Study of Mitochondrial Disorders, Department of Pediatrics and Inherited Metabolic Disorders, First Faculty of Medicine, Charles University and General University Hospital in Prague, 128 08 Prague, Czech Republic

**Keywords:** coenzyme Q10, multiple system atrophy, atypical parkinsonism, plasma, lymphocytes

## Abstract

Multiple system atrophy (MSA) is generally a sporadic neurodegenerative disease which ranks among atypical Parkinson’s syndromes. The main clinical manifestation is a combination of autonomic dysfunction and parkinsonism and/or cerebellar disability. The disease may resemble other Parkinsonian syndromes, such as Parkinson’s disease (PD) or progressive supranuclear palsy (PSP), from which MSA could be hardly distinguishable during the first years of progression. Due to the lack of a reliable and easily accessible biomarker, the diagnosis is still based primarily on the clinical picture. Recently, reduced levels of coenzyme Q10 (CoQ10) were described in MSA in various tissues, including the central nervous system. The aim of our study was to verify whether the level of CoQ10 in plasma and lymphocytes could serve as an easily available diagnostic biomarker of MSA. The study reported significantly lower levels of CoQ10 in the lymphocytes of patients with MSA compared to patients with PD and controls. The reduction in CoQ10 levels in lymphocytes correlated with the increasing degree of clinical involvement of patients with MSA. CoQ10 levels in lymphocytes seem to be a potential biomarker of disease progression.

## 1. Introduction

Parkinsonian syndrome (PS) is clinically characterized as a combination of two or more of the following symptoms: slowness of voluntary movement (bradykinesia), involuntary increased muscle tone (rigidity), tremor and/or postural instability. PS could be caused by neurodegeneration or by secondary etiology including normal pressure hydrocephalus or vascular disease. In the case of neurodegeneration, the most frequent cause is Parkinson’s disease (PD) (in at least 95% of cases). The rest are classified as so-called atypical PSs including Lewy body disease (LBD), progressive supranuclear palsy (PSP), corticobasal syndrome and multiple system atrophy (MSA).

MSA is a sporadic, adult-onset, progressive neurodegenerative disorder clinically manifested by a combination of autonomic dysfunction, pyramidal signs, parkinsonism (in the case of the MSA-P variant) and/or cerebellar involvement (in the case of MSA-C) [1].

The histopathologic basis of MSA involves the deposition of a pathologically conformed protein called alpha-synuclein in the central nervous system (mainly in the form of glial cytoplasmic inclusions in oligodendrocytes), but also in peripheral tissues such as muscle, kidneys, liver, lungs, heart, testes, blood vessels, cerebrospinal fluid (CSF), blood plasma, platelets, lymphocytes and red blood cells [2,3,4,5,6,7,8,9,10].

MSA pathogenesis has not yet been satisfactorily elucidated [11]. Multifactorial influence is assumed. The main hypotheses for the development of the disease include the role of inflammation, neurotoxicity, malfunction of the ubiquitin–proteasome system, iron accumulation and insulin resistance, but also the role of oxidative stress and mitochondrial dysfunction with an impaired respiratory chain (RC) function in complexes II and III. This malfunction of the RC has been found in the white matter, oligodendrocytes, neurons and peripheral tissues, e.g., in fibroblasts [12,13,14,15,16], but not in the substantia nigra and platelets [17]. Alpha-synuclein overexpression appears to inhibit the RC complex [11,18,19], which, in combination with the ability of alpha-synuclein to disrupt mitochondrial membrane integrity, may lead to increased production of reactive oxygen species (ROS) [20,21,22].

However, disruption of mitochondrial functions and the resulting increase in oxidative stress have been described also in other neurodegenerative PSs including idiopathic PD (with a deficit of respiratory chain complex I activity) and PSP [23]. Several genes involved in the pathophysiology of PD have been identified, namely PINK1 and Parkin, as playing roles in mitochondrial dysfunction [24,25]. PS as a direct consequence of mitochondrial damage has been observed in the MPTP (1-methyl-4-phenyl-1,2,3,6-tetrahydropyridine) toxin-induced model of PD which inhibits the RC complex I. A similar mechanism has been hypothesized in the Guadeloupean endemic parkinsonism after the consumption of annonacin [25,26,27].

MSA, as well as PSP and LBD, can be difficult to distinguish from idiopathic PD and secondary PSs, especially in the first years of disease progression. All these diseases could be initially expressed only as mild parkinsonism and may even respond satisfactorily to levodopa treatment.

The early prominent autonomic dysfunctions (orthostatic hypotension, urinary incontinence and constipation) are typical for MSA. However, these symptoms may be present also in the other PSs. Therefore, MSA is often detected only at the time of rapid progression with early instability, recurrent falls, severe dysarthria, dysphonia, stridor or the diminishing effect of levodopa treatment [1,28]. The abovementioned clinical picture remains the main diagnostic tool as there is still a lack of an early, readily available biomarker. Consequently, the diagnosis could be delayed by several years [29]. The main paraclinical method that helps us in the differential diagnosis is MRI (magnetic resonance imaging), but the sensitivity of MRI findings is not satisfactory [30]. Other potential biomarkers are being intensively studied. One of them could be CoQ10, whose significantly decreased levels have been previously described in the plasma, liquor and cerebellum of MSA patients [31,32,33].

CoQ10 is an endogenously produced, lipid-soluble molecule with a ubiquitous presence in tissues. In the body, CoQ10 switches between its reduced and oxidized forms as ubiquinol/ubiquinone, with normal plasma concentrations between 0.5 and 2 µmol/L [34]. Its main function consists of participating in the mitochondrial respiratory chain, where it acts as an electron carrier from complexes I and II to complex III. It thus ensures the proper function of oxidative phosphorylation, intracellular antioxidation and, among other things, contributes to the biophysical properties of cell membranes. In addition to endogenous production, it is absorbed from food.

Mutations in the *COQ2* gene, encoding the coenzyme Q2 4-hydroxybenzoate polyprenyltransferase which is essential for CoQ10 production, have been described in patients with MSA [35]. However, measurements of CoQ10 levels have not shown a dependence on the MSA phenotype, genetic *COQ2* mutation nor, so far, on the disease stage [32].

The aim of our study was to verify whether CoQ10 levels in plasma and lymphocytes could serve as an easily available diagnostic biomarker of MSA. Measured CoQ10 levels in MSA were compared with groups of patients with PD and PSP, as well as with healthy controls (HC). The resulting values of CoQ10 were compared with the degree of clinical impairment to verify the possible correlation of clinical deficit with CoQ10 levels. When compared to HC and groups of patients with PD, we found significantly reduced CoQ10 levels in lymphocytes in the MSA group of patients, which negatively correlated with the degree of clinical impairment. Plasma CoQ10 levels in MSA were lower compared to HC but did not differ between patient groups.

## 2. Materials and Methods

### 2.1. Subjects

Our cohort included 20 patients (10 F, 10 M, age between 50 and 80 years) with MSA who met the revised clinical diagnostic criteria [1]. The patients’ clinical status was objectified using specific scales of the NNIPPS-PPS (Natural History and Neuroprotection in Parkinson Plus Syndromes Parkinson Plus Scale) [36] and UMSARS (United Multiple System Atrophy Rating Scale) [37] (Table S1).

We compared MSA patients with healthy controls (n = 23, 16 F, 7 M, age between 50 and 75 years) (Table S2) and with patients diagnosed as clinically probable for PSP (n = 21, 7 F, 14 M, age between 53 and 85 years) (Table S3) and PD (n = 21, 12 F, 9 M, age between 38 and 83 years) (Table S4) [38]. The PSP patients’ clinical status was assessed using the NNIPPS-PPS and PSP rating scale [39] (Table S3). Sex, age, time of disease progression and degree of clinical disability were recorded for all patient groups (Tables S1–S4).

Clinical examination, as well as blood collection and processing of biological material, was performed based on the informed consent of the patients and approved by the Ethics Committee of the General University Hospital in Prague (19/19 S-IV 17 January 2019) and the 1st Faculty of Medicine of Charles University.

### 2.2. Samples

On the day of examination, venous blood was collected for lymphocyte separation, plasma and biochemical examination of total cholesterol. Patients taking statins were excluded because of a possible negative influence on CoQ10 synthesis by HMG CoA (3-hydroxy-3-methylglutaryl coenzyme A) reductase blockade.

### 2.3. Sample Preparation

Plasma samples were prepared from Li/Heparin-treated blood by centrifugation at 800 g at room temperature, immediately frozen and stored at −80 °C until analysis. Lymphocytes were isolated from 7 mL of EDTA-treated blood by differential centrifugation on Ficoll gradient as described in Askeland [40]. The final pellet of the cells was stored at −80 °C until the next step. Frozen pellets were homogenized in a Potter–Elvehjem homogenizer (Bellco glass, Vineland, NJ, USA) in STE medium (10 mM Tris-HCl, pH 7.4; 250 mM sucrose; 1 mM EDTA) at 4 °C. A cell homogenate was used for CoQ10 determination as described in [41]. Protein concentration in the homogenate was analyzed by Lowry method [42]. Citrate synthase (CS) activity was determined according to Srere [43]. Protein content and CS activity were used for the normalization of the Q10 value. Cholesterol was determined by the standard photometric method.

Total CoQ10 content was analyzed by HPLC (HPLC 20 prominence system, Shimadzu, Japan) with UV detection according to Mosca [44].

All chemicals were purchased from Sigma-Aldrich (St. Louis, MO, USA). Q10 standard was obtained from TANAKA (TANAKA, Tokyo, Japan).

### 2.4. Statistical Methods

Differences between HC and particular patient groups were analyzed using a two-sample *t*-test. Differences among patient groups were subject to a one-way ANOVA; *p*-values less than 5% were considered statistically significant. Analyses were conducted using an R statistical package version 4.2.2.

The tested parameters (except cholesterol in plasma) were not dependent on age and sex. The differences between the monitored groups were analyzed.

## 3. Results

### 3.1. Plasma Levels

**Figure 1:**
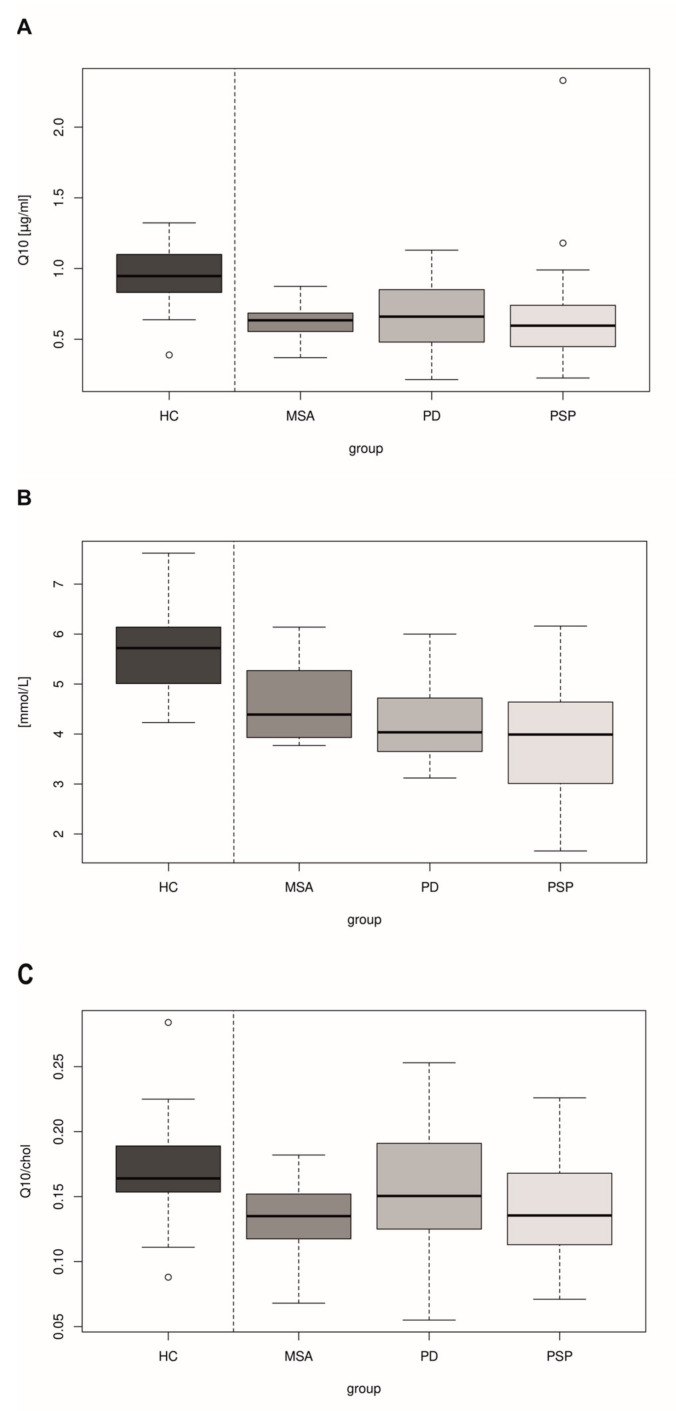
(**A**). Decreased plasma CoQ10 in the patient groups with multiple system atrophy (MSA, *N*= 20), Parkinson’s disease (PD, *N* = 21) and progressive supranuclear palsy (PSP, *N* = 21) in comparison with healthy controls (HC, *N* = 20). *t*-test, comparison of HC and MSA (*p* = 0.0000), comparison of HC and PD (*p* = 0.0002), comparison of HC and PSP (*p* = 0.0131). Test of difference between variables (ANOVA) *p* = 0.8667. (**B**). Decreased cholesterol in the patient groups with multiple system atrophy (MSA, *N* = 20), Parkinson’s disease (PD, *N* = 21) and progressive supranuclear palsy (PSP, *N* = 21) in comparison with healthy controls (HC, *N* = 20). *t*-test, comparison of HC and MSA *p* = 0.0005, comparison of HC and PD *p* = 0.0000, comparison of HC and PSP *p* = 0.0000. Test of difference between variables (ANOVA) *p* = 0.0398. (**C**). Decreased plasma CoQ10 in the patient groups with multiple system atrophy (MSA, *N* = 20) and progressive supranuclear palsy (PSP, *N* = 21) in comparison with healthy controls (HC, *N* = 20) when normalized to total cholesterol (chol). Patients with Parkinson’s disease (PD) did not show a statistically significant reduction in comparison with HC. *t*-test, comparison of HC and MSA *p* = 0.0032, comparison of HC and PD *p* = 0.4003, comparison of HC and PSP *p* = 0.0412. Test of difference between variables (ANOVA) *p* = 0.2997.

The results of the study showed a significant reduction in CoQ10 in the plasma of all patient groups when compared to healthy controls (Figure 1A, Table S5). The decreases in plasma CoQ10 levels in patients with MSA did not reach statistical significance compared to patients with PD and PSP (Figure 1A). The levels of cholesterol in plasma were significantly lower in all patient groups compared to healthy controls (Figure 1B). Significant differences in the parameter CoQ10/chol (level of coenzyme Q10 normalized to cholesterol level) in plasma were found between MSA and PSP groups compared to controls, but not between the individual MSA, PSP and PD groups (Figure 1C).

### 3.2. Lymphocyte Levels

**Figure 2:**
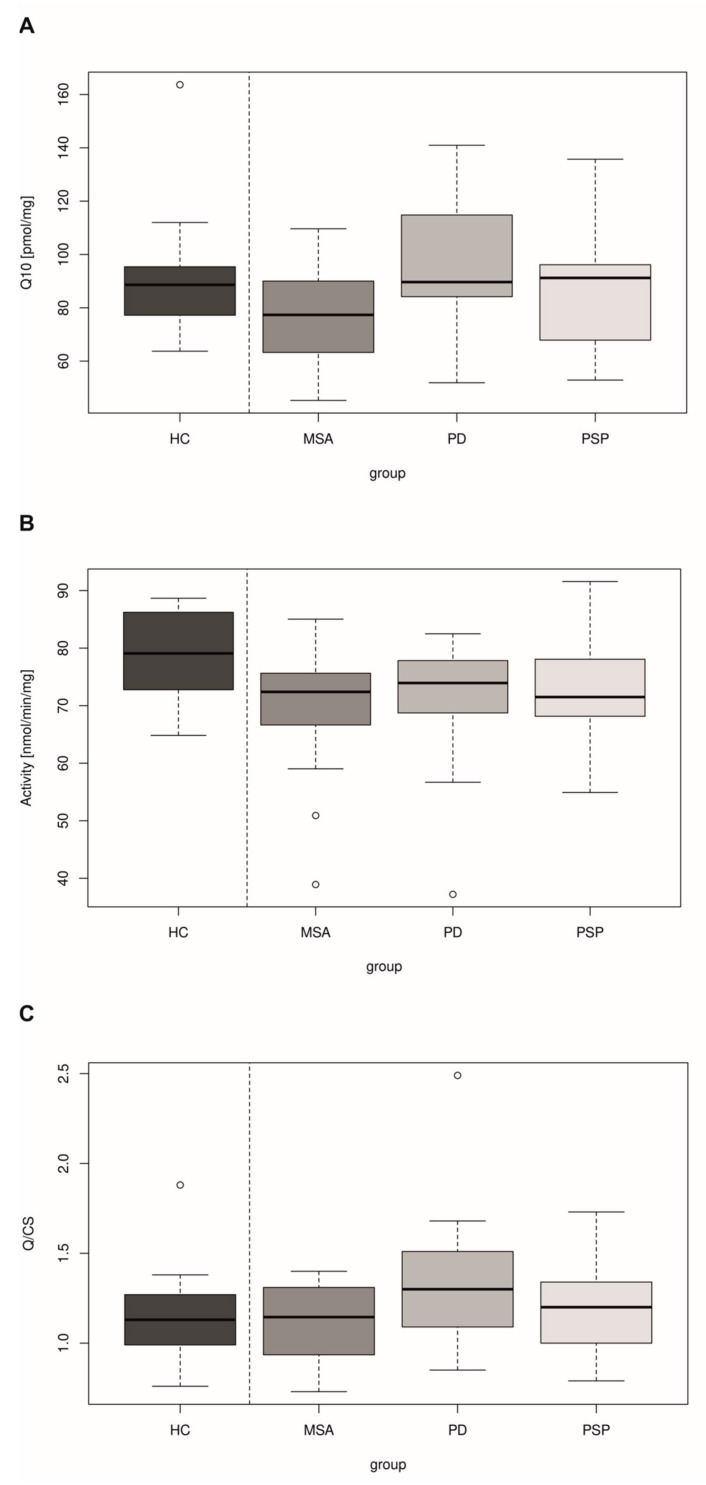
(**A**). Total coenzyme Q10 content in isolated lymphocytes from peripheral blood in healthy controls (HC, *N* = 20) in comparison with patients with multiple system atrophy (MSA, *N* = 20), Parkinson’s disease (PD, n = 21) or progressive supranuclear palsy (PSP, n = 21). Decreased CoQ10 is evident in patients with MSA compared to HC (*t*-test, *p* = 0.0397). A test of difference between variables (ANOVA) showed a significant difference between MSA and PD patients (*p* = 0.0329). (**B**). Activity of citrate synthase (CS) in isolated lymphocytes. Activity of CS was significantly lower in patients with multiple system atrophy (MSA, *N* = 20), Parkinson’s disease (PD, *N* = 21) and progressive supranuclear palsy (PSP, *N* = 21) in comparison with healthy controls (HC, *N* = 20). *t*-test, comparison of HC and MSA (*p* = 0.0047), comparison of HC and PD (*p* = 0.0098), comparison of HC and PSP (*p* = 0.0068). Test of difference between variables (ANOVA) *p* = 0.8621. (**C**) CoQ10 normalized to CS in isolated lymphocytes from peripheral blood (Q/CS) of patients with multiple system atrophy (MSA, *N* = 20), in comparison with patients with Parkinson’s disease (PD, *N* = 21), progressive supranuclear palsy (PSP, *N* = 21) and healthy controls (HC, *N* = 23). A significant difference (ANOVA) was found between the MSA and PD groups (*p* = 0.0356).

**Figure 3:**
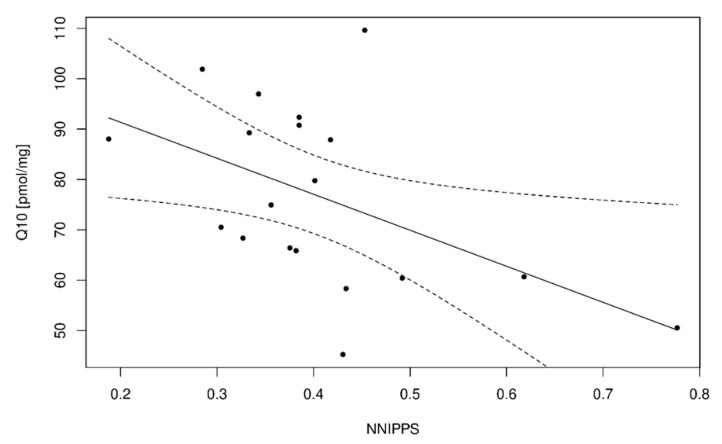
Inverse correlation between total coenzyme Q10 levels in isolated lymphocytes from peripheral blood of patients with multiple system atrophy (MSA, *N* = 20) and clinical stage of the disease (measured by Natural History and Neuroprotection in Parkinson Plus Syndromes–Parkinson Plus Scale) NNIPPS (*N* = 19). Test of association between variables (Spearman’s correlation coefficient, ρ = −0.4695): *p* = 0.0425. To illustrate the association between the variables, the regression line (the solid line) accompanied by its confidence bands (the two dashed lines) is shown.

The study reported significantly lower levels of CoQ10 in the lymphocytes of patients with MSA compared to controls and patients with PD (*p* = 0.0397, *p* = 0.0329, respectively) (Figure 2A). The levels of CoQ10 in lymphocytes in the MSA group inversely correlated with the severity of clinical impairment, using a specific scale for atypical Parkinson’s syndromes, the NNIPPS-PPS (*p* = 0.0425) (Figure 3).

A significant difference was found between the MSA and PD groups (*p* = 0.0356) in the parameter CoQ10/CS (level of coenzyme Q10 normalized to activity of citrate synthase) in lymphocytes (Figure 2C), whereby CS activity was significantly reduced in all three analyzed groups of patients and no difference of CS was found between the patient groups (Figure 2B).

## 4. Discussion

### 4.1. CoQ10 in Plasma

When compared to healthy controls, we found a reduction in plasma CoQ10 levels in MSA patients as well as in the PD and PSP groups (Figure 1A). The reduction in plasma CoQ10 levels in MSA is in accordance with previous studies [32,45]. The reduction in plasma CoQ10 levels in all patient groups probably corresponds to the impaired oxidative metabolism in Parkinson’s disease and other Parkinsonian syndromes [46,47]. However, when comparing patient groups, differences between CoQ10 levels did not reach statistical significance (Figure 1A). Therefore, plasma CoQ10 levels do not appear to be able to distinguish between individual Parkinsonian syndromes. These results may also be influenced by potential fluctuations in plasma CoQ10 levels depending on the actual requirement or oral intake as we suppose that plasma CoQ10 is only of transport significance [48].

When measuring CoQ10 levels, the value of current total cholesterol was considered because of the known association of CoQ10 and cholesterol in their partially shared biosynthesis pathway. In addition, lipoproteins act as CoQ10 carriers in the circulation, with the potential to form conjugates [49,50]. In our patient groups, the highest levels of cholesterol were found in MSA patients (median 4.390 mmol/L) and then PD patients (median 4.035 mmol/L); the lowest levels were shown in the PSP group (median 3.990) (Tables S1–S3). All patient groups showed lower cholesterol levels when compared to HC (Figure 1B), indicating possible changes in lipid metabolism. However, from these results, we are not able to conclude whether patient groups showed lower cholesterol levels because of a disturbance in its biosynthesis or because of higher metabolic demands with a secondary decrease. Decreased plasma cholesterol in patients with Parkinsonian syndrome may correspond to neurodegenerative involvement of the central nervous system [51]. Disturbances in lipid metabolism related to aberrant functions of alpha-synuclein have been previously described in PD. According to some papers, higher cholesterol and lipid levels may be associated with a better prognosis [52,53,54,55].

Patients taking statins that block HMG-CoA reductase, a key enzyme in cholesterol synthesis, and CoQ10 were also excluded from the study [56]. Our patients were treated with levodopa, but it was proven that serum CoQ10 levels and the Q10/chol ratio were not influenced by levodopa treatment in PD patients [57].

### 4.2. Lymphocytes from Peripheral Blood Are Valuable Biological Material for Analysis of Mitochondrial Parameters

In our study, the CoQ10 levels were simultaneously measured in the plasma and lymphocytes of peripheral blood. Peripheral blood cells are an important noninvasive biological material for the analysis of mitochondrial energy metabolism, bringing us valuable information about the current state of patients. Lymphocytes and platelets from peripheral blood are commonly used for the monitoring and diagnostics of respiratory chain disorders [58], but also for the analysis of secondary damage of mitochondrial energy metabolism in other diseases such as AD, HD and PD [40,59,60]. Bioenergetic parameters in the blood cells may reflect the situation in tissues with high energy demand. Duncan et al. found a close association between the CoQ10 level in skeletal muscle and WBC, but not with CoQ10 in plasma in CoQ10-deficient patients [61]. Decreased levels of CoQ10 in lymphocytes as well as in fibroblasts were also proven to be a cause of respiratory chain dysfunction in mitochondrial encephalopathies [62]. Lymphocytes have also been used in PD, with proof of significantly reduced activity in respiratory chain complexes I and IV [63]. The importance of CoQ10 levels in the lymphocytes of peripheral blood in protecting against stress-induced apoptosis has been described [64].

### 4.3. CoQ10 in Lymphocytes

In our study, a significant reduction in CoQ10 in lymphocytes in MSA was found (*p* = 0.0397) when compared to PD (Figure 2A). Compared to healthy controls, a significant decrease in CoQ10 was detected only in patients with MSA, not in those with PD and PSP. A significant reduction in CoQ10 (*p* < 0.001) in patients (both in MSA-P and MSA-C) was already previously observed in fibroblasts in comparison with controls [65].

According to the results, lymphocyte levels represent a more stable and reliable indicator of the CoQ10 total amount than the CoQ10 plasma level. Under physiologically normal conditions, blood cells or organs may regulate their CoQ10 content independently of the environmental supply [66].

The finding of insufficient CoQ10 levels in the lymphocyte pool may have severe consequences for the function of the respiratory chain in patients’ lymphocyte mitochondria and may be related to impaired antioxidant function, low ATP production and thus increased proapoptotic activity of lymphocyte cells [51]. CoQ10 also acts as a phenolic antioxidant in its reduced form, called ubiquinol. In this form, it inhibits protein and DNA oxidation, inhibits the peroxidation of cell membrane lipids and has only a direct antiatherogenic effect [67]. Decreased levels of CoQ10 may contribute to MSA pathogenesis due to decreased electron transport in the mitochondria and an increased vulnerability to exogenous oxidative stress with the production of reactive oxygen species [68,69].

In patients with CoQ10 deficiency, quinone-dependent activities in lymphocytes (complexes I and III, complexes II and III, glycerol-3-phosphate and complex III) were in the lowest absolute control values [61]. RC disturbance has already been described in MSA tissues. Foti et al. found decreased complex II/III and Q10 deficiency in MSA cerebellar white matter [16]. Blin et al. demonstrated a deficit in the muscle mitochondrial complex I activity (less than 30% of the mean) in MSA patients compared to age-matched controls [70]. CoQ10 is physiologically reduced in complex I of the RC by NADH:CoQ reductase activity. In the case of the impairment of this reductase, a sufficient proton gradient cannot be provided. A defect in this reductase with an impaired respiratory chain was described in the striatum of patients with PD [71,72].

### 4.4. Possible Causes of Differential Q10 in Parkinson’s Disease Patients

In general, decreased CoQ10 levels could be caused by the primary defects in CoQ10 biosynthesis or due to the known secondary effects of statin pharmacotherapy. Specifically in PS patients, CoQ10 deficiency could have an association with the aforementioned common V343A variant in the *COQ2* gene in sporadic MSA [73], mutation in the *NUS1* gene [74] (influencing the dolichol biosynthetic pathway that shares common biosynthetic steps with Q10), a hypothesized dysregulation of the specialized lipid metabolism involved in myelin synthesis and maintenance in MSA [75], or unknown individual genetic backgrounds (e.g., different mtDNA haplogroups) [76]. The decrease in lymphocyte CoQ10 levels compared to healthy controls only in MSA, but not in PD and PSP, may be caused by a higher degree of stability of intracellular coenzyme Q10 levels. Thus, a decrease in lymphocyte CoQ10 may become apparent only with a more significant decrease and depletion of total CoQ10 which is seen in MSA patients. In PD and PSP, the reduction in CoQ10 is unlikely to reach threshold levels that would be reflected intracellularly.

### 4.5. Correlation of CoQ10 Levels with the Clinical State

Using the specific scale for atypical PSs, the NNIPPS-PPS, decreased levels of CoQ10 in lymphocytes correlated with increasing levels of clinical disability in MSA patients including motor, cognitive, behavioral and functional involvement (*p* = 0.0425) [36] (Figure 3). Based on this observation, it is plausible that CoQ10 could play a role as a secondary marker of disease progression in the future. These results follow a published study demonstrating a correlation between low levels of CoQ10 and clinical disability measured by the UMSARS in patients with MSA-C [45]. In our study, the difference between the clinical subtypes of MSA (MSA-C/MSA-P) could not be assessed due to the small number of MSA-C patients in our cohort and in the general Caucasian population.

Our work does not specify whether the low CoQ10 levels are a cause or a consequence of clinical deterioration. The fact that peroral supplementation of CoQ10 has not produced convincing results, despite its isolated successes, may lead to the idea that the deficit of CoQ10 in MSA could be just a secondary problem [77,78,79,80]. On the other hand, limited therapeutic results of CoQ10 supplementation have also been observed in mitochondrial encephalopathies caused by primary CoQ10 deficiency. Therefore, the therapeutic failures could also be caused by the limited absorption and bioavailability of CoQ10 for limited permeability through the blood–brain barrier [50].

It is necessary to prove our results in a larger patient group as there is still the need for a reliable biomarker. This is related to the fact that MSA continues to be underdiagnosed in clinical practice. As mentioned above, MSA can mimic PD in the early stages and is often diagnosed only after several years of progression. However, diagnostic delay may lead to inappropriate management of the disease and an associated poorer quality of life [81,82,83].

### 4.6. Limitations of the Study

The main limitation of the study was the small cohorts of MSA and PSP patients. This was mainly due to the low prevalence of the disease in the population and its rapid progression.

Patients with definitive diagnoses verified by autopsy findings were not included in our study due to the absence of a longitudinal follow-up; however, the diagnosis was not changed in the years following the termination of study recruitment. Future research directions could also be highlighted.

## 5. Significance of Differential Q10 in Parkinson’s Disease Patients and Conclusions

To the best of our knowledge, this is the first work investigating levels of CoQ10, not only in plasma but also originally in lymphocytes. Based on our results, it seems that the decrease in CoQ10 plasma levels confirms the disturbance of oxidative metabolism in Parkinsonian syndromes compared to healthy controls. However, it is not a sufficiently specific marker to distinguish between Parkinsonian syndromes. Conversely, the measurement of CoQ10 levels in lymphocytes seems to be a reliable biomarker in the differential diagnosis of PSs. Moreover, finding a correlation between clinical decline and the decreased levels of CoQ10 suggests that CoQ10 levels in lymphocytes could be useful in monitoring the disease’s progression. Further work is needed towards the identification of early biomarkers for MSA, not only for better diagnostic aspects but also to implement future disease-modifying therapies at an early stage.

## Data Availability

Data is contained within the article and Supplementary Materials.

## Supplementary Materials

The following supporting information can be downloaded at: https://www.mdpi.com/article/10.3390/antiox12122104/s1. Table S1. The results of plasma and lymphocytes levels of coenzyme Q10 in patients with multiple system atrophy and the clinical scales. Table S2. The results of plasma and lymphocytes levels of coenzyme Q10 in healthy controls. Table S3. The results of plasma and lymphocytes levels of coenzymeQ10 in patients with progessive supranuclear palsy and the clinical scales. Table S4. The results of plasma and lymphocytes levels of coenzyme Q10 in patients with Parkinson’s disease. Table S5. *p*-values for comparison of variables between patients groups and controls and within patient groups. Differences between healthy controls and particular patient groups were analyzed using a two-sample *t*-test. Differences among patient groups were subject to a one-way ANOVA. *p*-values less than 5% were considered as statistically significant. Analyses were conducted using an R statistical package version 4.2.2. MSA—multiple system atrophy, PD—Parkinson’s disease, PSP—progressive supranucelar palsy, HC—healthy controls, chol—cholesterol.

## Abbreviations

PS—Parkinsonian syndrome; PSP—progressive supranuclear palsy; MSA—multiple system atrophy; PD—Parkinson’s disease; LBD—Lewy body dementia; CoQ10—coenzyme Q10; MPTP—1-methyl-4-phenyl-1,2,3,6-tetrahydropyridine; EDTA—ethylenediaminetetraacetic acid; RC—respiratory chain; NNIPPS-PPS—Natural History and Neuroprotection in Parkinson Plus Syndromes Parkinson Plus Scale; UMSARS—United Multiple System Atrophy Rating Scale.
