# Supplementary material for: Coenzyme Q10: A Biomarker in the Differential Diagnosis of Parkinsonian Syndromes
Source: Antioxidants (Basel). 2023 Dec 12;12(12):2104. doi: 10.3390/antiox12122104 (PMC10740444; doi:10.3390/antiox12122104)
Supplement: Supplementary file 1 [file antioxidants-12-02104-s001.zip › antioxidants-2731949-supplementary.pdf]

Coenzyme Q10: A Biomarker in the Differential Diagnosis of Parkinsonian Syndromes

Tereza Bartošová, Jiří Klempíř and Hana Hansíková

Supplementary data

Table S1. The results of plasma and lymphocytes levels of coenzyme Q10 in patients with multiple system atrophy and the clinical scales.

Patient.	Sex	Age [y]	Years of progression	NNIPPS PPS***	UMSARS*** **	P-Q10 [μg/ml] *	P-chol [mmol/l]	P-Q10/chol	L-Q10/CS	L-Q10 [pmol/mg]**	L-CS [nmol/min/mg]
P1	M	70	5.5	106	57	0.71	3.9	0.182	1.36	96.97	71.16
P2	F	60	5	134	85	0.54	3.87	0.14	0.74	58.33	79.27
P3	F	63	3	133	52	0.7	4.39	0.159	0.73	45.26	61.81
P4	M	79	7.5	101	56	0.57	3.93	0.145	1.01	68.35	67.52
P5	F	66	5.5	191	87	0.8	nd	nd	0.85	60.66	71.13
P6	M	65	11	240	93	0.37	nd	nd	1.3	50.55	38.92
P7	F	73	5	129	73	0.57	4.66	0.122	1.16	87.87	75.75
P8	M	55	7	94	51	0.66	4.38	0.151	0.97	70.51	72.38
P9	M	61	5	152	95	0.4	3.93	0.102	1.19	60.4	50.91
P10	F	57	2	nd	nd	nd	nd	nd	1.07	80.43	75.5
P11	M	79	3	118	66	0.67	5.27	0.127	0.95	65.85	69.53
P12	F	67	3,5	119	62	0.67	3,82	0.175	1.4	92.36	65.77
P13	F	79	8	88	34	0.46	3.77	nd	1.35	101.9	75.52
P14	M	49	4.5	124	66	0.66	4,3	0.153	1.35	79.75	59.03
P15	F	74	4.5	132	69	0.798	6.14	0.13	1.32	109.64	82.94
P16	M	55	4	119	65	0.394	5.76	0.068	1.13	90.76	80.19
P17	M	55	4.5	103	56	0.572	5.06	0.113	0.88	74.92	85.03
P18	F	53	3.5	38	31	0.874	5.79	0.15	1.18	88.04	74.62
P19	M	54	4	103	56	0.634	4.9	0.13	1.18	89.26	75.44
P20	F	62	5	116	67	0.596	5.37	0.11	0.92	66.38	72.41
**N**		20	20	19	19	19	17	17	20	20	20
**Average**		63.8	5,05	123.15	64.26	0.61	4.66	0.13	1.1	76.9	70.24
**Median**		62.5	4.75	119	65	0.634	4.39	0.135	1.145	77,335	72.395
**Q1**		55	3.625	103	56	0.54	3.915	0.11525	0.9275	61.9575	66.2075
**Q2**		62.5	4.75	119	65	0.634	4.39	0.135	1.145	77.335	72.395
**Q3**		72.25	5.5	133	73	0.7	5.32	0.1525	1.615	90.385	75.6925

*Normal plasma levels of CoQ10 in plasma: 0,5- 2 [μg/ml]; **normal levels of CoQ10 in lymphocytes 70-160 [pmol/mg]; *** range of NNIPSS-PPS is 0-309 points; ***** range of UMSARS is 0-109 points; nd (not done), Q10- coenzyme Q10, CS- citrate synthase, chol – plasma cholesterol, L- lymphocytes, P- plasma.

Table S2. The results of plasma and lymphocytes levels of coenzyme Q10 in healthy controls

Healthy control	Sex	Age [y]	P-Q10 [µg/ml]*	P-chol [mmol/l]	P-Q10/chol	L-Q10/CS	L-Q10 [pmol/mg]**	L-CS [nmol/min/mg]
H1	M	51	1.098	5.74	0.191	1.17	88.02	74.98
H2	M	73	1.201	4.23	0.284	1.30	86.74	66.88
H3	F	63	1.102	6.13	0.180	1.00	88.91	88.67
H4	F	60	1.217	5.98	0.204	1.27	112	88.35
H5	F	65	0.997	5.72	0.174	0.95	75.97	80
H6	F	50	0.84	5.4	0.156	1.02	89.91	58.48
H7	F	62	1.015	6.03	0.168	1.03	88.61	85.91
H8	F	53	0.800	4.55	0.181	0.81	69.7	86.56
H9	F	50	0.683	5.46	0.117	0.14	78.43	68.61
H10	M	74	1.04	4.63	0.225	1.27	104.33	82.44
H11	M	58	0.703	6.34	0.111	0.81	63.71	78.87
H12	F	59	1.202	7.62	0.158	0.90	71.21	79.09
H13	F	52	0.896	5.46	0.164	1.33	94.94	71.6
H14	F	66	0.96	6.15	0.156	1.24	80.12	64.84
H15	F	57	0.947	6.55	0.145	1.38	102.77	74.41
H16	F	60	0.822	5.11	0.161	1.08	92.04	85.22
H17	F	55	1.241	6.16	0.201	1.27	92.43	73.02
H18	F	53	0.924	6.1	0.151	1.88	163.65	87.15
H19	M	54	0.864	5.48	0.158	1.13	95.83	84.06
H20	F	54	1.323	6.89	0.192	1.24	97.66	78.49
H21	M	52	0.698	4.82	0.145	0.98	71.2	72.51
H22	M	51	0.389	4.43	0.088	0.76	66	87.25
H23	F	58	0.916	4.91	0.187	1.11	79.27	71.4
**N**		23	23	23	23	23	23	23
**Average**		57.82	0.95	5.67	0.169	1.09	89.28	77.77
**Median**		57	0.947	5,72	0.161	1.11	88.02	78.87
**Q1**		52	0.822	4.91	0.151	0.95	75.97	71.6
**Q2**		57	0.947	5.72	0.164	1.11	88.61	78.87
**Q3**		62	1.102	6.15	0.191	1.27	95.83	85.91

*Normal plasma levels of CoQ10 in plasma: 0,5- 2 [µg/ml]; **normal levels of CoQ10 in lymphocytes 70-160 [pmol/mg]; *** range of NNIPSS-PPS is 0-309 points; ***** range of UMSARS is 0-109 points; nd (not done), Q10- coenzyme Q10, CS- citrate synthase, chol – plasma cholesterol, L- lymphocytes, P- plasma.

Table S3. The results of plasma and lymphocytes levels of coenzymeQ10 in patients with progessive supranuclear palsy and the clinical scales.

Patient	Sex	Age	Years of progression	NNIPPS /309	PSP RATING/ 100	P-Q10 [µg/ml]*	P-chol [mmol/ l]	P-Q10/ chol	L-Q10/CS	L-Q10 [pmol/mg] **	L-CS [nmol/min/ mg]
P1	M	74	4,5	103	39	0.63	2.98	0.211	0.92	60.07	64.96
P2	F	69	2	119	56	0.74	nd	nd	0.92	62.54	68.16
P3	M	67	2	62	23	2.33	nd	nd	1.56	107.17	68.91
P4	F	64	5	120	50	0.64	nd	nd	1.19	86.47	72.53
P5	F	73	5	164	64	1.18	nd	nd	1.34	96.15	71.5
P6	M	62	5.5	142	59	0.48	nd	nd	0.95	52.89	55.44
P7	M	81	4	157	70	0.24	3.04	0.079	1.73	135.72	78.36
P8	F	70	1.5	104	24	0.53	3.2	0.166	1.33	106.91	80.23
P9	M	74	1	153	66	0.4	2.97	0.135	1.51	82.7	54.91
P10	M	53	4	48	18	0.99	4.38	0.226	1.41	96.01	68.3
P11	F	77	4.5	110	49	0.815	6.16	0.132	1.11	85.6	77.01
P12	M	76	6	101	43	0.226	1.66	0.136	1.31	82.7	63
P13	M	76	8	131	69	0.63	4.66	0.135	1.20	91.2	75.73
P14	M	75	2	90	53	0.448	3.07	0.146	0.79	61.73	78.07
P15	M	85	6.5	53	19	0.596	4.31	0.14	1.13	94.92	84.33
P16	M	75	4.5	58	21	0.455	2.61	0.17	1.48	93.55	63.08
P17	F	82	2	73	29	0.822	4.62	0.18	1.28	94.94	74.24
P18	M	66	2	51	28	0,5	4.94	0.1	1.23	98.45	80.25
P19	F	70	4.5	131	58	0.621	4.94	0.126	1.00	67.87	68.2
P20	M	77	9.5	149	63	0.264	3.71	nd	0.84	59.35	70.62
P21	M	68	8.5	157	63	0.386	4.27	nd	1.148613	105.19	91.58
**N**		21	21	21	21	21	16	14	1	21	21
**average**		72.09	4.4	108.38	45.9	0.62	3.84	0.148	1.21	86.7	71.8
**median**		74	4.5	110	49.5	0.596	3.99	0.138	1.2	91.2	71.5
**Q1**		67.5	2	67.5	26	0.424	2.995	0.1305	0.975	65.205	66.56
**Q2**		74	4.5	110	50	0.596	3.99	0.138	1.2	91.2	70.5
**Q3**		76.75	5.75	145.5	63	0.7775	4.65	0.1725	1.375	97.3	78.215

*****

range of PSP RATING scale is 0-100 points

*Normal plasma levels of CoQ10 in plasma: 0,5- 2 [µg/ml]; **normal levels of CoQ10 in lymphocytes 70-160 [pmol/mg]; nd (not done), Q10- coenzyme Q10, CS- citrate synthase, chol – plasma cholesterol, L- lymphocytes, P- plasma

Table S4. The results of plasma and lymphocytes levels of coenzyme Q10 in patients with Parkinson's disease.

Patient	Sex	Age	Years of progress ion	P-Q10 [µg/ml]*	P-chol [mmol/L]	P-Q10/ chol	L- Q10/CS	L-Q10 [pmol/mg]**	L-CS [nmol/min/mg]
P1	F	38	4	0.851	4.75	0.179	1.41	115.46	81.88
P2	F	73	10	0.457	3.62	0.126	1.57	116.32	77.2
P3	F	59	4	0.687	6	0.115	1.09	84.62	77.83
P4	M	65	7	0.86	4.5	0.191	1.54	121.32	79.02
P5	M	80	14	0.409	3.12	0.131	1.17	85.27	72.66
P6	M	67	6	0.553	3.68	0.15	1.46	115.74	79.21
P7	M	63	11	0.945	nd	nd	1.30	89.51	68.74
P8	M	47	5	0.48	3.75	0.128	0.85	70.18	82.49
P9	M	60	3	0.628	4.16	0.151	1.22	89.65	73.23
P10	F	53	1	0.677	5.42	0.125	1.09	84.46	77.23
P11	F	56	2	0.215	3.91	nd	0.98	67.74	68.49
P12	M	72	5	0.679	3.55	0.191	0.93	75.76	81.52
P13	M	83	1	0.31	3.41	0.091	0.88	64.93	73.94
P14	F	71	13	0.505	nd	nd	1.39	51.88	37.21
P15	F	55	14	0.52	4.2	0.124	1.31	97.75	74.86
P16	F	64	5	0.76	4.57	0.166	1.68	114.79	68.32
P17	M	50	11	0.93	3.68	0.253	1.51	108.85	72.14
P18	F	69	8	1.13	4.72	0.239	2.49	140.95	56.69
P19	F	73	17	0.66	3.65	0.181	1.68	97.94	58.38
P20	F	67	8	1.09	4.81	0.227	1.21	91.24	75.26
P21	F	65	15	0.39	nd	nd	1.21	84.16	69.44
**N**		21	21	21	18	17	21	21	21
**average**		63.3	7.8	0.65	4.19	0.16	1.33	93.73	71.55
**median**		65	7	0.66	4.04	0.1585	1.3	89.65	73.94
**Q1**		55.5	4	0.4685	3.65	0.1255	1.09	79.96	68.615
**Q2**		65	7	0.66	4.16	0.1151	1.3	89.65	73.94
**Q3**		71.5	12	0.8555	4.75	0.191	1.525	115.125	78.425

*Normal plasma levels of CoQ10 in plasma: 0,5- 2 [µg/ml]; **normal levels of CoQ10 in lymphocytes 70-160 [pmol/mg]; nd (not done), Q10- coenzyme Q10, CS- citrate synthase, chol – plasma cholesterol, L- lymphocytes, P- plasma.

Table S5. P-values for comparison of variables between patients groups and controls and within patient groups. Differences between healthy controls and particular patient groups were analyzed using a two-sample t-test. Differences among patient groups were subject to a one-way ANOVA. P-values less than 5% were considered as statistically significant. Analyses were conducted using an R statistical package version 4.2.2. MSA – multiple system atrophy, PD – Parkinson's disease, PSP – progressive supranucelar palsy, HC – healthy controls, chol- cholesterol.

	**Comparison**			
	**HC and MSA**	**HC and PD**	**HC and PSP**	**Patient Groups**
Q10 plasma	0.0000	0.0002	0.0131	0.8667
Q10 lymphocytes	0.0397	0.4927	0.6864	0.0329
Q10/CS lymphocytes	0.6505	0.0414	0.3176	0.0406
CS lymphocytes	0.0047	0.0098	0.0068	0.8621
Q10/chol plasma	0.0032	0.4003	0.0412	0.2997
Cholesterol	0.0005	0.0000	0.0000	0.0398
